# Exceptional intrarenal pseudomembranous

**DOI:** 10.11604/pamj.2019.32.214.18385

**Published:** 2019-04-29

**Authors:** Souhail Regragui, Gabriel Stoica

**Affiliations:** 1 Urology Departement, CHIC Alencon Mamers, Alencon, France; 2 Urology B Departement, CHU Ibn Sina, Rabat, Morocco

**Keywords:** Pseudomembranous, renal, pyelotomy

## Image in medicine

We report the case of a 51-year-old patient, known as hypertensive and type 2 diabetic, admitted to emergency for acute pyelonephritis. She suffered from low back pain in a feverish context. A Uro-scanner showed the presence of a 12mm pyelic renal calculus and an calycal calculus of 6mm diameter responsible for a moderate dilation of left pyelocalictic cavities. First, we performed drainage with a double J probe. Then, in a second step, the left ureteroscopy allowed partial laser fragmentation. The presence of a suspect soft magma prompted us to stop the procedure. After performing a hydatid serology that has returned negative, a laparoscopic left pyelotomy allowed the progressive externalization of the suspect magma. It presented with a greenish, fibrinous, semi-solid shell completely molding the pyelon and the pyelocalicielles cavities. The introduction of the flexible cystoscope through a trocar at the end of the procedure allowed us to check the complete cleaning of the excretory cavities. The anatomopathological study is back in favor of a weakly eosinophilic acellular material with some polyhedral crystals and some inflammatory elements. There were no signs of malignancy. The patient did not present any postoperative complication and did not present a recurrence.

**Figure 1:**
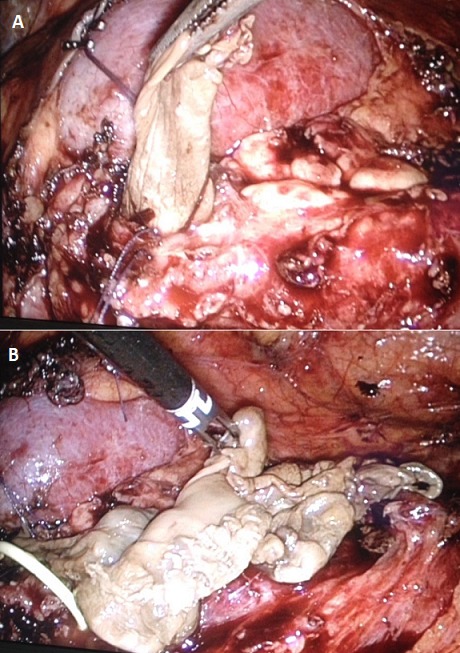
A) extraction of the pseudomembranous from the kidney; B) final aspect of the pseudomembranous

